# Modeling the Effect of Physical Activity on Obesity in China: Evidence from the Longitudinal China Health and Nutrition Study 1989–2011

**DOI:** 10.3390/ijerph14080844

**Published:** 2017-07-27

**Authors:** Tao Zhang

**Affiliations:** School of Public Administration, Macao Polytechnic Institute, Macao 999078, China; Taozhang@ipm.edu.mo; Tel.: +853-85-996-281

**Keywords:** obesity, physical activity, causality, regression, model, China

## Abstract

Although physical activity has been widely recognized as an important influential factor in determining the risk of obesity, the results in the existing literature empirically examining such issue are mixed. Especially for China, relevant studies are rarely found. One aim of this study is to test the direction of effects between obesity and physical activity. It uses longitudinal data to investigate the relationship and causality between physical activity and obesity for both children and adults in China. The longitudinal data and dynamic panel model used here can yield more solid results than the other studies employing cross-sectional data, particularly considering strict endogeneity and self-selection. It is discovered that obesity does not affect children’s physical activity but that obese children are more sedentary. For adults in China, physical activity can significantly reduce the weight, but not in the opposite direction.

## 1. Introduction

As a global epidemic, obesity appears to stem from physical inactivity, whereas opposite evidence shows obesity probably inducing physical inactivity. Though it is widely recognized that physical activity (PA) is significantly correlated with obesity, such association cannot be easily interpreted as a causal relationship, particularly when only using cross-sectional data in empirical analysis. Sisira et al. [1] examined the effect of PA on adult obesity using panel data from Canada and found the significant causality mechanism between PA and obesity. Littman et al. [2], Gordon-Larsen et al. [3], Hankinson et al. [4], Ekelund et al. [5], and Williams [6] discovered the significantly negative association between PA and obesity risk using longitudinal data. By contrast, some other studies proposed that the relationship between PA and obesity over time is unclear and insignificant [7,8,9]. These results make the empirical relationship between PA and weight gain mixed and unclear. In addition, although the negative association between PA and obesity is always easily found in the literature using cross-sectional data [10,11,12,13], the nature of cross-sectional data shows that such significant correlation cannot be simply interpreted as a causal relationship [14,15]. Hill had proposed some important factors which might help judging a causal relationship [16]. Temporality is considered to be an important characteristic. This study will use dynamic model to test the causal relationship between PA and obesity, in which temporality is sufficiently examined. Furthermore, some other factors, such as significance, strength, consistency, and specificity will also be explored in the analysis. 

Hitherto, available literature concerning China’s public health fails to account for the causality between obesity and PA. Nearly no observed studies investigating the relationship between obesity and PA in China use dynamic panel data in order to clearly explain the causality mechanism between PA and obesity, while also controlling other plausible factors. This study attempts to address this problem by employing nationally representative longitudinal data from the China Health and Nutrition Study (CHNS) from 1989 to 2011. Therefore, the objective of this study is to examine the relationship between PA and body mass index (BMI) in China, while accounting for the dynamic adjustments. Both the children and adults are considered when estimating the causation between PA and obesity. Due to the possibly distinct mechanism existing in the association, the treatment needs to be different. Subsequently, panel Granger causality tests will be employed to investigate the plausible reciprocal causation between different physical activities and BMI for children in China, whereas, a dynamic panel model for multi-variables will be estimated for adults.

## 2. Materials and Methods

Generally, obesity is conceptualized as maintaining a BMI higher than 30, whereas in China according to Zhou [17] it is proposed that a BMI of 28 or above should be defined as obesity. Chou et al. [18] suggests that the energy intake and expenditure in each period will determine the weight gain. Subsequently, physical activity is of considerable importance in understanding the risk of obesity. Moreover, according to Sisira et al. [1], in addition to energy balance over time, the modifiable risk factors for obesity include a number of observable and unobservable idiosyncratic individual characteristics. In order to understand the empirical causality mechanism between PA and obesity, some other influential factors such as socio-economic and demographic variables, are also considered as they may influence the prevalence of obesity significantly in the past decades in China [19]. As the model employed here using longitudinal data, the contribution of unobservable factors to some extent can be captured by the unobserved individual-specific heterogeneity term or simply called individual effect. In this case, Hausman test will be used to choose the random-effects or fixed-effects estimators. Since fixed-effects estimation allows the correlation between individual effects and explanatory variables, based on the F-test and Hausman test, this study concerns weigh heavily on the fixed-effects estimators logically. The exogeneity of explanatory variables also should be considered for the consistency of panel estimators [20]. Therefore, to examine the plausible existence of such feedback effect, this study employs the method proposed by Wooldridge [20]. Since this study emphasizes the relationship between PA and BMI, the lagged value of PA should also be considered. This study specifies the lags of PA for two time periods in the model as:

BMI*_i,t_* = α + ηBMI*_i,t_*_−1_ + ρ(PA)*_i,t_*_−2_ + τ(PA)*_i,t_*_−1_ + λ(PA)*_i,t_* + β’X*_i,t_* + u*_i_* + ε*_i,t_*. (1)

where *i* represents individual observation and *t* indicates time for each time period. BMI*_it_* is the body mass index for individual *i* at time *t*. PA*_it_* represents the physical activity by individual *i* at time *t*. X*_it_* is a vector of observed factors of individual *i* at time *t*. u*_i_* represents the individual-specific unobserved heterogeneity term for each individual *i*. And, ε*_it_* is the error term for each individual *i* at time *t* with the assumption of zero expectation and constant variance. The vector of observed variables contains gender, age, household income, educational status, and dummy for location of residence (rural area or urban area).

However, Nickell [21] proposed that fixed-effects estimation would yield biased estimators. To address this problem and obtain consistent estimates, the estimation method proposed by Arellano and Bond [22], Arellano and Bover [23] and Baltagi [24] is used. This method requires the removing of individual effects by taking first differences or forward orthogonal deviations. In this case, the autocorrelation test should be used for the difference operators.

As the effect of different physical activities on obesity probably differs by different ages, this study conducts separate analyses to investigate the distinct influence of children and adults. Due to the unbalance of panel data and the different effect of socio-demographic variables on obesity, this study separates observations of population into two groups for different analyses. Children aged 6 to 18 are included in one group and the adults in another group. Since some demographic variables of children are not suitable for the regression estimation discussed above, the estimation of Equation (1) is only applied to adults. Another method specifically designed to examine the causality relationship between PA and BMI or obesity is used for children. In addition, the causality test was chosen for children because the panel data of these explanatory variables for children are seriously unbalanced. If we use dynamic regression, it will not yield any result for such reason. The method first proposed by Granger [25], also called Granger Causality test, provides the approach to test the question of whether A causes B or B causes A. If two-way causation does exist, then we can say A Granger causes B and simultaneously B Granger causes A. Although it should be noted that Granger causality is not totally equal to the common use of the term “causality”, it does measure the precedence and information content from cohort and time-series perspective. This is an important predetermining factor when we are going to define a causation relationship. Based on the basic Granger causality test, Dumitrescu and Hurlin [26] suggested an approach to measure the Granger causality for panel data with the assumption of all coefficients to be different across cross-sections. Moreover, it also can be measured by the totally opposite assumption that all coefficients are invariant across all cross-sections. This study employs such approach to examine the Granger causality of different physical activities and obesity for children from 6 to 18 years old.

**Figure 1:**
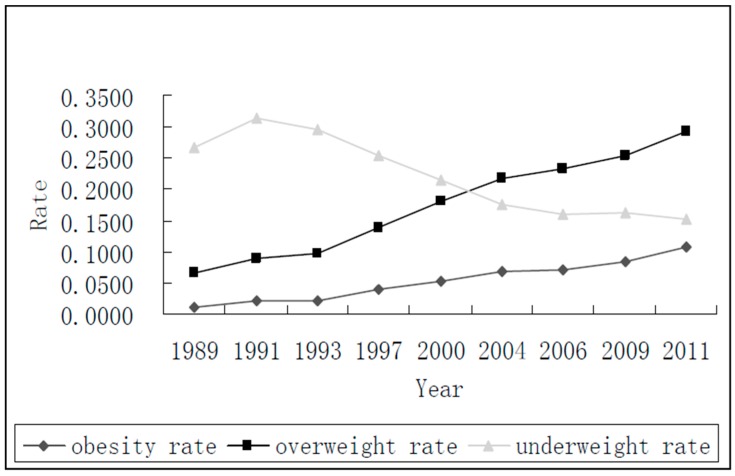
Obesity rate over time.

The longitudinal data used in this study are taken and processed from the 9 cycles of the cohort CHNS from 1989 to 2011. This is an international collaborative project conducted by the Carolina Population Center at the University of North Carolina and the National Institute for Nutrition and Health at the Chinese Center for Disease Control and Prevention (CCDC). The CHNS collected the individual information using a multistage, random cluster process to draw a sample of over 30,000 individuals in 15 provinces in China. This study divides the sample into three groups by ages for separate analyses in order to avoid undue influence of different age spans. As the variables specifically related to obesity are interested in this study, the variables of obesity rate, overweight rate and underweight rate over time will be respectively investigated. Figure 1 depicts the obesity rate, overweight rate and underweight rate from 1989 to 2011.

The obesity rate increased from 1% in 1989 to 10.74% in 2011 steadily and continuously. With the sharp growth of economy in these decades, the prevalence of obesity in China’s population was also increased. The overweight rate also increased greatly from 6.5% in 1989 to 29.27% in 2011, whereas underweight rate decreased from 31.34% in 1991 to 15.125 in 2011. Here, according to World Health Organization (WHO) and Asian-Pacific Consensus Statement, the recommended BMI cut points for overweight and underweight are 25 and 18.5 respectively. 

**Figure 2:**
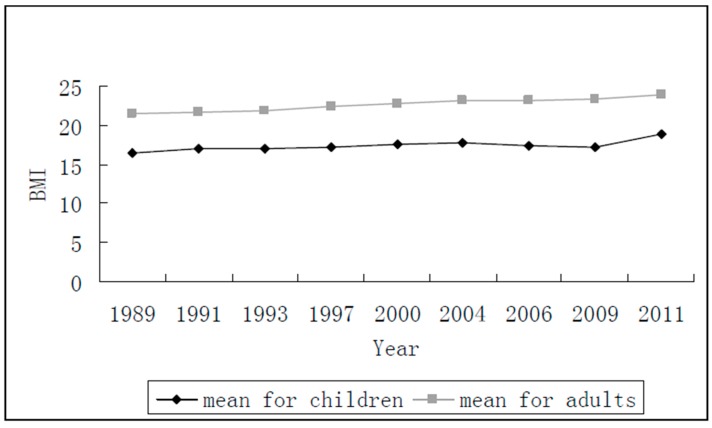
Body Mass Index (BMI) over time.

Because this study investigates the different age spans separately, the summary statistics of BMI for the children aged 6–18 and adults, respectively, were examined. Figure 2 describes the means of BMI for adults and children over time. It is clear that the means of BMI for both children and adults increased from 1989 to 2011. The mean of BMI for children only had small changes from 1989 to 2008, but it increased greatly from 2009 to 2011, whereas, the mean of BMI for adults increased continuously from 21.51 in 1989 to 23.80 in 2011. Six variables were chosen to denote the physical activity of children in the Grange causality test. They were assessed as follows: A dummy variable ‘Participating in PA’ represents whether a child participates in the physical exercises usually. A value of 1 indicates the child usually participating, whereas 0 means not. This dummy variable depended on self-reported data and the judgment of the respondents in the survey. The continuous variable ‘PA before or after school’ indicates the time of a child engaging in physical exercises per week before or after school, which is measured in minutes. The variable ‘PA in school’ denotes the hours of physical exercises per week a child doing in school. The variable ‘Watching television (TV)’ represents the time of a child watching TV or video per week before or after school, which is measured in minutes. The variable ‘Reading or writing’ denotes the time of a child reading or writing per week before or after school, which is also measured in minutes. The variable ‘Walking’ measures the time a child walking from home to school in minutes. Table 1 provides the means of above variables over time. The variables of ‘Watching TV’ and ‘Reading or writing’ were only collected in two cycles of cohort in 1997 and 2000. Although two-period panel is too short to provide enough information for longitudinal regressions, it can still give some useful implications when we use Ganger causality test. ‘Participating in PA’, ‘PA in school’, and ´Walking’ were collected in the six cycles of cohort, while ‘PA before or after school’ was collected in the four cycles of cohort.

Table 1 displays the means of six variables denoting physical activity or inactivity of children over time. The overall trend of the time of walking from home to school over 14 years shows to be upward from about 30 min in 1997 to 41 min in 2011. This indicates that the students walking to school spend more time on the way. This may increase their energy expenditures. The time doing PA in school also roughly holds an increasing trend from 3.75 h in 1997 to 6.43 h in 2011. ‘Participating in PA’ is a dummy variable, and subsequently the mean of it, in fact, presents the proportion of children usually participating in the physical exercises. It is increased from 1997 to 2011, whereas decreased from 2009 to 2011. The time doing PA before or after school decreased from 2004 to 2006, whereas continuously increased from 50.8 min per week in 2006 to 58.2 min per week in 2011. The time watching TV and the time reading or writing both displayed a declining trend in two periods. Particularly for the time reading and writing, it was reduced from 451 to 210 min in only three years, indicating a significant decrease in writing and reading for students after or before school. 

Variable of education measures the number of years the adults receiving the formal education. From Table 2, it clearly has an increasing trend in 20 years. Household income increased greatly from 4243.57 Yuan RMB in 1989 to 44,186.12 Yuan RMB in 2011. Nominally, the household income was improved more than 10 times in 23 years. 

The means of explanatory variables for longitudinal regressions are displayed in Table 2. The variable of education measures the number of years the adults receiving the formal education. From Table 2, it clearly has an increasing trend in 20 years. Household income increased greatly from 4243.57 Yuan RMB in 1989 to 44,186.12 Yuan RMB in 2011. Nominally, the household income was improved more than 10 times in 23 years.

PA denotes the time of physical activity an adult doing per week, measured in minutes. It is only collected in the six cycles of cohort continuously. The mean of PA changed shiftily over time. It increased from 1997 to 2004, and then decreased. As expected for longitudinal data, the mean of age increased continuously over time. The change of age was not totally coincident with periodical change of year due to the unbalanced panel of data used here. The regional dummy denotes the location of adults, while 0 denotes urban area and 1 denotes rural area. The mean of such dummy, in fact, presents the proportion of people located in the rural area. From Table 2, it shows that this proportion decreased slightly from 0.69 in 1989 to 0.66 in 2011. Gender also is a dummy variable with 0 denoting male and 1 denoting female. It nearly was not changed over time. 

## 3. Results

### 3.1. Results of Granger Causality Test for Children

Table 3 displays the results of six causality tests for six variables denoting children’s physical activity or inactivity. Since causality test is estimated by bivariate regressions, it can examine two-way causation relationship between the BMI and the PA variable. For the relationship between ‘Participating in PA’ and BMI, the null hypothesis that ‘Participating in PA’ does not causally influence BMI is rejected significantly, whereas the opposite direction is not rejected. This plausibly presents that the change of ‘Participating in PA’ is a cause for the change of BMI over time but the change of BMI does not cause the change of ‘Participating in PA’ longitudinally. The two-way causality or reciprocal causation is not found in this test. Children exercising on a regular basis are in better shape. The null hypotheses that ‘PA in school’ does not causally influence BMI and BMI does not causally influence ‘PA in school’ are both not rejected, indicating that there is no significant causal relationship between them. The null that ‘PA before or after school’ does not causally influence BMI is significantly rejected, showing that the children’s PA outside school influences BMI and therefore body weight greatly. However, the null that BMI does not causally influence ‘PA before or after school’ is not rejected, again indicating that the children will not participate in PA outside school because of their high BMI or body weight. As for ‘Reading or writing’ and BMI, it shows that both directions of causation are not statistically significant. However, the relationship between BMI and ‘Watching TV’ is interesting. The null hypothesis that ‘Watching TV’ does not influence BMI is accepted, whereas higher BMI does causally influence ‘Watching TV’. This may result from the fact that the obese or overweight children will be less physically active and thus spend more time watching TV or video. The null hypothesis that BMI does not causally influence ‘Walking from home to school’ is significantly accepted, whereas the children walking a longer distance to school will be in better physical shape and have a healthy weight since the null hypothesis denoting the inexistence of their causation is significantly rejected. One important thing noted again is that Ganger causation is not totally equal to the common use of the word especially when there are some other unobserved factors which may influence both of them. Therefore, it should be interpreted cautiously as possible unobserved influential variables may exist. The causality test for BMI and adults’ PA is not reported in Table 3 since the relationship between them will be investigated clearly by panel regressions in the next subsection. However, this test is also executed and the result showed that the adults’ PA causally influences the BMI but not in the opposite direction. Generally, it can be observed that obesity will not induce children to participate in PA to reduce weight. However, they will be more inactive due to obesity. However, for adults, it was shown that PA can significantly reduce body weight whereas the impact of BMI on PA is unclear. This relationship is only statistically unclear, which may be caused by the mutual offset from the two opposite way effects. Some obese adults will be more inactive while some others may conduct their exercises to reduce the weight. In addition to the above PA variables listed in Table 3, the school grade of children is considered as the possible influential factor. The result shows that BMI cannot influence the school grade while the school grade will significantly influence BMI. This may be come from the fact that the children as getting older in the higher school grade will have higher BMI. Furthermore, the association between the mother’s weight and children’s BMI is also estimated. However, it yields insignificant results in both directions. This may be due to the seriously unbalanced panel data of the mother’s weight.

### 3.2. Results of Longitudinal Regressions for Adults

To have solid statistical results for panel regressions, the fixed effects estimation using static fixed effects regression is performed firstly. Then, the F-test for the existence of all fixed effects was performed. The estimated F-statistic is 5.77 with freedom at 13,719. Therefore, the null hypothesis that all the fixed effects are jointly equal to zero was significantly rejected. This result solidly tells us that unobserved individual effects should be considered, and thus the pooled ordinary least squares may yield inconsistent estimates. In addition, for carefully comparison, the random effects estimation was also executed. The test for the random effects is also performed. The null of no random effects is significantly rejected again with the estimated χ^2^ statistic at 15,806.56. After examining the existence of individual effects, the Hausman test is used to test the correlation between individual effects and other explanatory covariates. The null that random effects estimation is consistent is significantly rejected with χ^2^ statistic at 357.02. Consequently, the regression with fixed effects should be employed in this study. The potential feedback problem is tested by the method proposed by Wooldridge [20]. The null hypothesis of strict exogeneity is not rejected with F-statistic at 0.02. In this case, the strict exogeneity assumption of longitudinal regression is satisfied, and thus Equation (1) can be estimated consistently. In addition, the autocorrelation test for BMI shows a significant serial autocorrelation, whereas the first difference of BMI nearly has not serial autocorrelation. This result combined with the panel unit root test indicates that the dynamic panel regression should be used here. Therefore, although the estimates of static panel regressions with fixed effects or random effects are also reported in Table 4, the dynamic panel regressions estimated by Equation (1) using the Arellano-Bond method proposed by Arellano and Bond [22], Arellano and Bover [23] and Baltagi [24] are more reliable.

The first explanatory variable is used to interpret the relationship between resident’s location and BMI in China. The random effects and fixed effects estimates show an insignificant and negative relationship between location dummy and BMI, whereas two dynamic panel regressions both yield a significant and positive coefficient. Such large difference between static regressions and dynamic regressions presents the importance of inclusion of the lagged BMI term in the model. Again, both the static fixed effects and random effects regressions give the positive and insignificant coefficients for gender dummy, while both dynamic regressions provide positive and highly significant estimates. In addition, the magnitudes of the coefficients of the static models are smaller than the estimates of both dynamic models. The positively significant estimate of the dynamic model for gender dummy shows that women in China gain weight easier than men. In four regressions, only the random effects model yields the significant coefficient for education status. Since the dynamic model should provide more consistent results, the unclear and insignificant association between education and BMI is more dependable. This result is obviously different from the previous cross-sectional studies suggesting a significantly negative association between education and BMI [27,28,29]. However, some other longitudinal studies using dynamic models also found an insignificant or weakly significant association between education and BMI [1].

Household income is positively significant for the random effects model and dynamic panel model using Equation (1). This shows that the increasing income could improve the prevalence of obesity in China from 1989 to 2011. As for age, all four models give positively significant estimates at 1% level. This can be easily interpreted that people will gain more weight due to decreasing metabolism as they age. In addition, with increasing age, adults will gain weight because of more sedentary activities for the pressure from society and their work. The coefficients of the lagged BMI in the dynamic models are both positively significant at 1% level. It is interesting to find that the regressions yield strongly different results when including the term of lagged BMI. The relationship between physical activity and BMI is the main emphasis of this study. Except for static fixed effects model, all the three others give negatively significant coefficients for contemporary PA, clearly showing that increasing PA would reduce the risk of obesity in the same period. The estimated coefficient of the PA lagged with one time cycle (PA_t−1_) using dynamic model with Equation (1) is also negatively significant at 5% level, indicating that the physical activity in the last period will also negatively affect the obesity in the current period. In addition, the estimated coefficient of PA_t−2_ shows to be statistically insignificant. The estimated constants for all four models are significant at 1% level. The null hypothesis that all the explanatory variables are jointly equal to zero is significantly rejected at 1% level for all four regressions by Wald test.

To examine the effect of BMI on the PA, a dynamic panel model using the Arellano-Bond method is also performed. All the other explanatory variables are also included in the regression. For simplification, the estimates of the other factors are not reported. We only need to take into account the effects of BMI and lagged BMI on PA. Both the estimates for the BMI and the lagged BMI are negatively insignificant. This result reinforces the result of causality test, and again indicates that PA is a Granger-causation for BMI but not in the opposite direction in China.

## 4. Discussion

From the results, it shows that, not specifically for reducing weight, China’s children who habitually do physical exercise are in better shape. As children’s physical activity outside school influences BMI greatly, more exercises after school should be given to China’s children. Watching TV or video will induce higher risk of obesity in children as they will be more inactive. Therefore, the parents should reduce the time children spend watching TV or video. As obesity will induce children to be more inactive, the parents should obligate these obese children to be more active. For adults, it shows that women in China gain weight easier than men. Thus, the government policy-makers should pay more attention to women while considering intervention. In addition, the increasing income improved the prevalence of obesity in China in the past decades. In conformity with many other studies, older people will gain weight because of their decreasing metabolism and more sedentary activities. Furthermore, PA would reduce the risk of obesity in the same period or in the next period. The results also indicate that PA can causally influence BMI but not in the opposite direction. This means that PA could really influence the BMI and therefore body weight in China’s adults.

## 5. Conclusions

This study uses longitudinal data to investigate the relationship and more importantly causality between PA and obesity. It can yield more robust estimates than those studies employing cross-sectional data, especially when accounting for endogeneity and self-selection problems. As understanding the association between PA and obesity has become a focal point of public health in China, the upward prevalence of obesity makes such longitudinal investigation more important. The sharp economic growth and subsequently an increase in the sedentary habits continue to result in a reduction in energy expenditure. Therefore, in recent decades, the BMI, the overweight rate, and the obesity rate are all increased significantly. Generally, both the economic growth and the shift of social environment are argued to be the main reason for the obesogenic trend of modern society. Although physical activity has been widely recognized as an important influential factor in determining the risk of obesity [30,31], the existing studies empirically exploring the association between physical activity and obesity are mixed and unclear. Furthermore, such empirical analysis for China is rarely observed, particularly when considering the advantage of longitudinal data. Therefore, the modeling of the relationship between PA and obesity using representative cohort data from 1989 to 2011 in China is an important contribution of this study.

In order to clearly find the causation between PA and obesity, this study attempts to use some statistical test. Although the Granger-causality test only can ascertain the casual relationship on the term of statistics, it still can tell us the causation relationship from the perspective of time-series or cohort effects. In this study, it is found that the obesity has not significant impact on children’s PA but the obese children will do activities more sedentary, such as watching TV and video. A variety of tests is employed to determine the model in estimating the relationship between the adult’s obesity and PA. Except for physical activity, other demographic factors and household income are also included in the model, which may coincidently influence the obesity. It shows that the dynamic panel regression estimated by the Arellano-Bond method should be chosen for the cohort data in this study. The result shows that for adults in China physical activity can significantly reduce the body weight whereas the effect in the opposite direction is unclear.

## Figures and Tables

**Table 1:** Means of six variables for Granger causality test over time.

Year	Walking (min)	PA in School (h)	Participating in PA (0/1)	PA Before or after School (min)	Watching TV (min)	Reading or Writing (min)
1997	30.02	3.75	0.07		405.55	451.07
2000	27.17	3.04	0.34		387.41	210.38
2004	37.60	3.76	0.35	54.85		
2006	39.09	5.97	0.34	50.81		
2009	37.72	5.53	0.37	52.22		
2011	40.86	6.43	0.34	58.20		

PA: physical activity.

**Table 2:** Explanatory covariates of panel regressions for adults.

Year	Education	Household Income	PA	Age	Urban/Rural	Gender
1989	6.81	4243.57		31.46	0.69	0.51
1991	5.22	4411.37		41.18	0.67	0.51
1993	6.03	6174.58		42.09	0.69	0.51
1997	6.23	11,754.65	890.99	43.58	0.65	0.50
2000	7.54	14,180.95	811.01	44.99	0.66	0.51
2004	8.71	18,288.92	1050.15	48.02	0.65	0.51
2006	8.58	21,801.42	996.72	49.27	0.66	0.52
2009	8.85	35,466.88	932.12	50.01	0.67	0.51
2011	9.09	44,186.12	939.48	51.68	0.66	0.52

**Table 3:** Pairwise Granger Causality Tests with 1 lag.

Null Hypothesis:	F-Statistic	Prob.
‘Participating in PA’ does not cause BMI	8.5446 ***	0.0035
BMI does not cause ‘Participating in PA’	1.9653	0.161
‘PA in school’ does not cause BMI	2.1007	0.1473
BMI does not cause ‘PA in school’	0.1818	0.6699
‘PA before or after school’ does not cause BMI	9.7411 ***	0.002
BMI does not cause ‘PA before or after school’	2.4027	0.1225
‘Reading or writing’ does not cause BMI	0.9261	0.3363
BMI does not cause ‘Reading or writing’	0.1201	0.7291
‘Watching TV’ does not cause BMI	0.4782	0.4894
BMI does not cause ‘Watching TV’	7.9869 ***	0.0048
‘Walking from home to school’ does not cause BMI	3.5197 **	0.0298
BMI does not cause ‘Walking from home to school’	0.1766	0.8382

Note: **, and *** give the coefficients’ significance indicated by estimated standard errors at 5% and 1% level, respectively. BMI: body mass index; TV: television.

**Table 4:** Panel regression results for adults.

	Static Fixed Effects Regression	Static Random Effects Regression	Dynamic Panel Regression without the Lagged PA	Dynamic Panel Regression with Equation (1)
Urban/Rural	−0.01633	−0.06740	4.27214 ***	5.40653 ***
Gender	0.12796	0.06986	10.44104 ***	9.94068 ***
Education	0.00498	0.04019 ***	0.00034	−0.00976
Household income (1000 Yuan)	0.00057	0.00466 ***	0.00032	0.00198 **
Age	0.10589 ***	0.05322 ***	0.03818 ***	0.03493 ***
PA	−0.00002	−0.00005 ***	−0.00006 ***	−0.00018 **
BMI(t−1)			0.46047 ***	0.38019 ***
PA(t−1)				−0.00034 **
PA(t−2)				0.00005
Constant	18.160 ***	19.710 ***	−12.149 ***	−10.930 **
Sigma_u	3.437	2.815		
Sigma_e	2.001	2.001		
Test of individual effects	F = 5.77 ***	χ^2^ = 15,806.56 ***		
Wald test for all variables	F = 305.96 ***	χ^2^ = 1218.46 ***	χ^2^ = 853.72 ***	χ^2^ = 734.79 ***

Note: The null hypothesis for F or Wald test is that the coefficients are jointly equal to zero. **, and *** give the coefficients’ significance indicated by estimated standard errors at 5% and 1% level, respectively.
